# The Full Model of the pMHC-TCR-CD3 Complex: A Structural and Dynamical Characterization of Bound and Unbound States

**DOI:** 10.3390/cells11040668

**Published:** 2022-02-14

**Authors:** Josephine Alba, Marco D’Abramo

**Affiliations:** Deptartment of Chemistry, Sapienza University of Rome, Piazzale Aldo Moro, 5, 00185 Rome, Italy

**Keywords:** molecular dynamics, T cell antigen receptor, biophysics, protein-membrane

## Abstract

The machinery involved in cytotoxic T-cell activation requires three main characters: the major histocompatibility complex class I (MHC I) bound to the peptide (p), the T-cell receptor (TCR), and the CD3 complex, a multidimer interfaced with the intracellular side. The pMHC:TCR interaction has been largely studied by means of both experimental and computational models, giving a contribution in understanding the complexity of the TCR triggering. Nevertheless, a detailed study of the structural and dynamical characterization of the full complex (pMHC:TCR:CD3 complex) is still missing due to a lack of structural information of the CD3-chains arrangement around the TCR. Very recently, the determination of the TCR:CD3 complex structure by means of Cryo-EM technique has given a chance to build the entire system essential in the activation of T-cells, a fundamental mechanism in the adaptive immune response. Here, we present the first complete model of the pMHC interacting with the TCR:CD3 complex, built in a lipid environment. To describe the conformational behavior associated with the unbound and the bound states, all-atom Molecular Dynamics simulations were performed for the TCR:CD3 complex and for two pMHC:TCR:CD3 complex systems, bound to two different peptides. Our data point out that a conformational change affecting the TCR Constant *β* (C*β*) region occurs after the binding to the pMHC, revealing a key role of this region in the propagation of the signal. Moreover, we found that TCR reduces the flexibility of the MHC I binding groove, confirming our previous results.

## 1. Introduction

**Figure 1:**
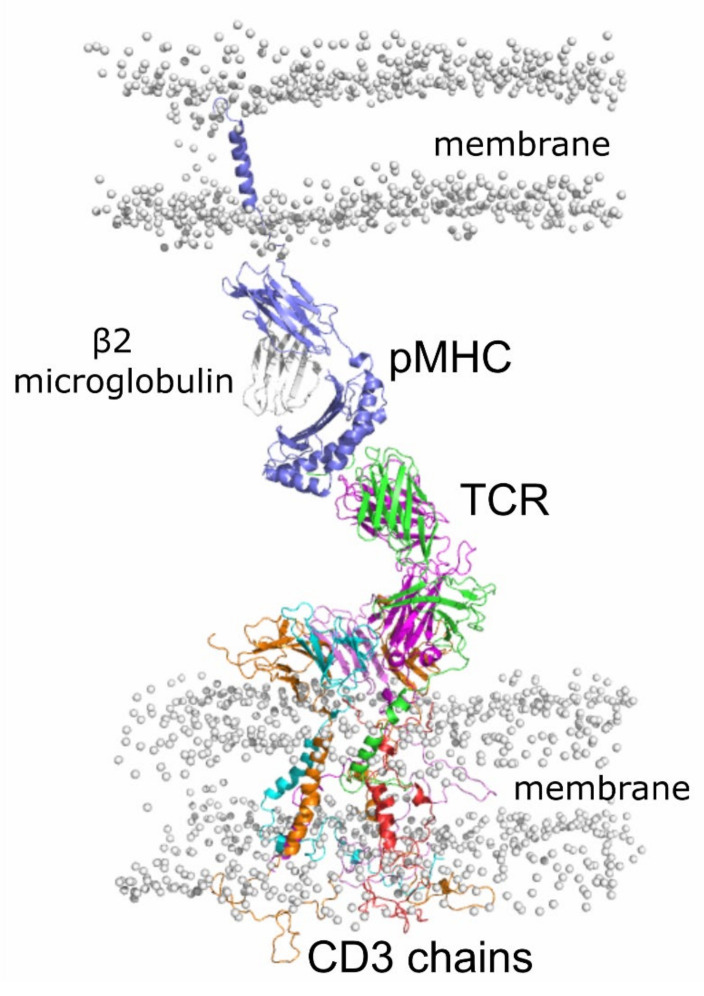
Model of the pMHC:TCR:CD3-chains in membrane. For clarity, only the lipid phosphate groups are shown (gray spheres) for the membrane.

**Figure 2:**
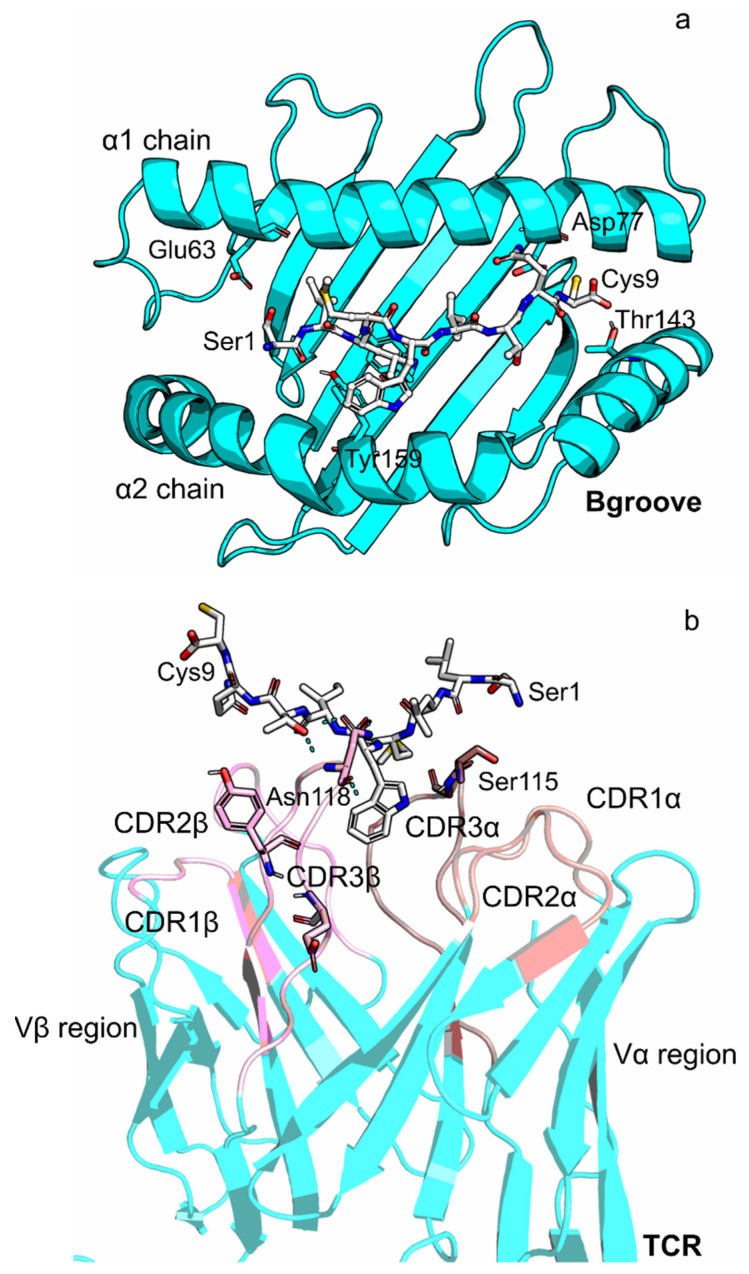
Zooming into the regions close to the peptide. Panel (**a**): MHC I binding groove (Bgroove, residues 1–180) bound to the peptide (cyan and gray respectively); panel (**b**): focus on the CDR loops of the TCR variable regions (pink) interfaced to the peptide (gray).

T cells play a crucial role in the adaptive immune response [1]. In fact, the TCR triggering is strictly connected to the T cell activation, and it is regulated by two main interactions: (i) the interaction between the peptide and the MHC I with the TCR, in the extracellular surroundings [2]; (ii) the interaction of the CD3 chains with the TCR in the intracellular environment [3] (Figure 1). The ability of TCR to recognize a huge number of peptides presented by the MHC I ensures a solid defense system from foreign pathogens [4]. MHC I is a protein complex formed by an *α* chain, which hosts the binding site for a nine/ten residue-long peptide, and a non-covalent bound *β*2 microglobulin [5] (Figure 1 and Figure 2a). pMHC interacts with TCR, a heterodimer composed of two transmembrane glycoprotein chains, named *α* and *β* [6]. The extracellular portion of each chain consists of two domains, the variable region (V) and the constant region (C). The Vα and the V*β* are formed by three complementarity-determining regions, the CDR-loops, which are directly involved in the interaction [7] (Figure 2b). The V*α* CDR1 and CDR2 loops are in close contact with the helices of the pMHC complex, while the V*β* CDR1 and CDR2 loops interact with the pMHC at the carboxy terminus of the bound peptide [8,9]. The binding of the TCR to the pMHC implies induced conformational changes especially in the V*α* CDR3 loop [9,10,11,12,13,14].

Previous studies have suggested that the interaction of CD3 and TCR *αβ*-chains propagates the pMHC-TCR-binding information to the ITAM regions [15,16,17,18,19,20,21]. However, although several models of the TCR–CD3 triggering have been proposed, this mechanism is still largely unknown [22,23].

Nevertheless, the determination of the first structure of the TCR interacting with the CD3 chains recently provided by cryo-EM [24] has given a chance to build the full construct of the CD3-TCR complex for in silico investigations. Using this structure, we present here the first complete computational model of the pMHC:TCR:CD3-chains in the presence of the lipid membrane (Figure 1), thus providing an extended structural-dynamic description of this complex with two different peptides bound to the MHC I.

## 2. Materials and Methods

### 2.1. Modeling of the pMHC:TCR:CD3-Chains Complex

To compare our findings with previous experimental [25,26] and computational works [27] (see Table 1), we chose the MHC I encoded by the allele HLA-A*02:01 (where HLA is the abbreviation for “Human Leukocyte Antigen”), bound to two different peptides: ESO9C (the tumor antigen NY-ESO157-165 fragment—SLLMWITQC 25,26]) and the mutated ESO4D (a.a. sequence SLLDWITQV [25]). Thus, we selected the 1G4 TCR, which is specific for the interaction between HLA-A*02:01 and ESO9C.

**Figure 3:**
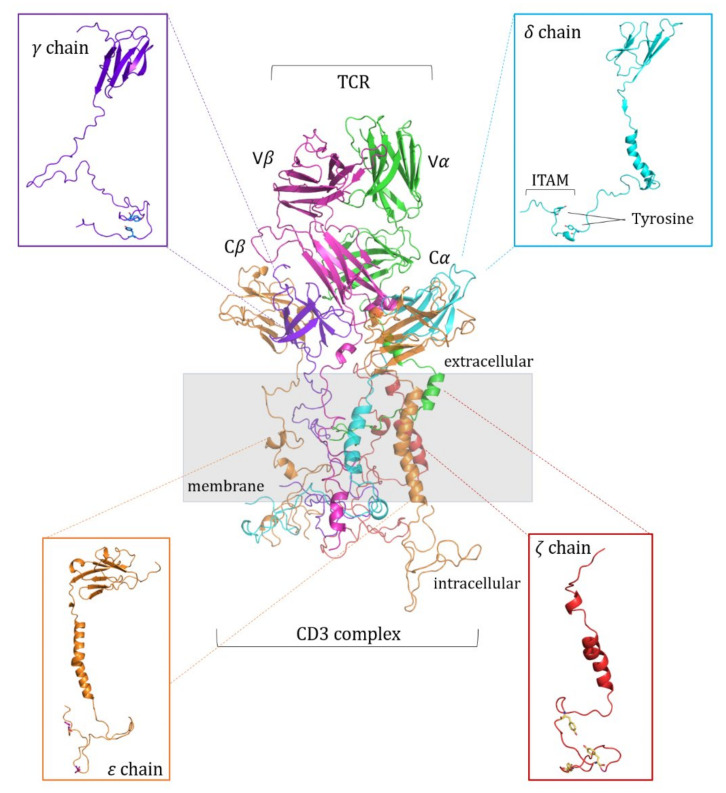
TCR:CD3 complex structure. The CD3 chains interact with the terminal region of the TCR (magenta and green) through non-covalent bonds. Each chain is magnified in the box. The ITAM region, containing the tyrosine residues, is indicated in the CD3*δ* chain box as reference.

From the cryo-EM structure of the TCR-CD3 complex (PDB ID: 6JXR, resolution of 3.7 Å), the missing parts of the CD3*γε*:CD3*δε*:CD3*ζζ* chains were built by means of Modeller Software package [28,29,30,31]. The best conformations—selected to avoid steric clashes—were then used to run the Molecular Dynamics simulations (Figure 3). To model the 1G4 TCR complex, the *αβ* chains provided by the corresponding crystallographic construct (PDB ID:2BNR) were aligned to the cryo-EM structure. The transmembrane helices were previously built by means of the Modeller Software [28], using the sequences as provided by the Uniprot database [32] (Uniprot entries P01848 and P01850 for the TCR *α* and *β* chains, respectively; Q9MY51 for the *α* chain of the HLA-A*02:01).

The resulting complexes were assembled, solvated with the TIP3P water model and minimized using GROMACS Software package version 2019.1 and the steepest descent algorithm [33]. Then, the solvent was removed, and the complex was inserted manually in the two membrane patches, using the VMD Software version 1.9.3. Lipids having a distance of 2Å from the protein were removed to avoid steric clashes. The membrane patches were previously built using the Charmm-Gui Membrane builder web server [34] with a heterogeneous lipid composition based on mammalian cell membrane [35] (1-palmitoyl-2-oleoyl-sn-glycero-3-phosphocholine (POPC) at 90%; phosphatidylinositol (4,5)-bisphosphate (PIP2) at 7%; 1-palmitoyl-2-oleoyl-sn-glycero-3-phospho-L-serine (POPS) at 3%). A recent study revealed that cholesterol inhibits TCR activation [36], and hence, it was excluded from our model.

Final systems of ~1,014,000 and 543,861 atoms were obtained for the bound and unbound states, respectively (see Table S1 for details).

### 2.2. Molecular Dynamics (MD) Simulations

The complexes were solvated with the TIP3P water model [37], adding Na+ and Cl- ions at a physiological concentration of 0.15 M (details about the numbers of water molecules and ions added are reported in Table S1). An energy minimization step was performed using the steepest descent algorithm without position restraints. Next, a series of equilibration steps were carried out: (1) an NPT equilibration lasting 2 ns to allow the packing of the lipids around the protein, with an integration time step of 0.2 fs; (2) an NVT equilibration of 4 ns, increasing the time step at 1 fs; (3) a production run lasting 1 us was run with a time step of 2 fs. The Parrinello-Rahman barostat [38] and the V-rescale thermostat [39] were used with semi-isotropic coupling with τp = 5 ps and τT = 0.1 ps, respectively. Considering the melting point of the lipid composition, the temperature was kept constant at 305 K. The particle mesh Ewald method [40] (cut-off of 1.2 nm) was used to treat the electrostatic interactions. A cut-off of 1.2 nm was used for the van der Waals interactions. The simulations were performed using the additive all-atom CHARMM36 force field [41], and the Gromacs Software version 2019.1 [33]. For each system, we performed a Molecular Dynamics simulation on the μs timescale (see Tables S2 and S3).

### 2.3. Structural Analysis

The root mean square deviation (RMSD) is a statistical measure of the average distance between a group of atoms, with respect to a reference structure:

(1) $$\mathrm{RMSD}=\sqrt{\frac{1}{N}\sum_{i=1}^{N}{\left|r_{i}\left(t\right)-r_{i}^{0}\right|}^{2}}$$

where *r_i_* (*t*) is the position of the atom *i* at the time *t*, *N* is the total number of atoms in the group considered and *r_i_*^0^ is the position of atom *i* in the reference structure. The RMSD calculation was performed on the alpha carbons, choosing the first frame of the simulation as reference.

The root mean square fluctuation (RMSF) is a statistical measure of the deviation between the position of the atom *i* (or a group of atoms, e.g., a residue), *r_i_* (*t*) and the initial structure *r_i_*^0^ considering the time interval *T*:

(2) $${\mathrm{RMSF}}_{i}=\sqrt{\frac{1}{T}\sum_{t_{j}=1}^{T}{\left|r_{i}\left(t_{j}\right)-r_{i}^{0}\right|}^{2}}$$

Concerning the analysis of the whole systems, the TCRs and the pMHCs, the RMSD and the RMSF were computed on all the alpha carbons, excluding the transmembrane regions and the CD3 chains. The first frame of each simulation was chosen as the reference structure. The RMSF calculations related to the CD3 chains were performed on the alpha carbons of each chain.

### 2.4. Essential Dynamics (ED)

The essential dynamics is a statistical method based on the principal component analysis (PCA) [42]. The covariance matrix of the atomic positions is built from the MD simulations on a selected group of atoms (usually C-alpha), to obtain a set of eigenvectors and eigenvalues describing the principal motion directions of the system. Thus, it is possible to represent the protein dynamics in a reduced space (essential subspace) defined by the eigenvectors. In the case of study, the essential subspace, describing the overall motion, is mostly confined within the first two eigenvectors. Combining two (or more) trajectories of different systems, it is possible to obtain common eigenvectors defining the subspace explored by the different proteins. Therefore, projecting the C-alpha on the first two eigenvectors (i.e., principal components), it is possible to compare the conformations sampled by the proteins during the simulation.

This analysis was carried out by means of the gmx covar and gmx anaeig tools as provided by GROMACS Software package 2019.1 [33].

### 2.5. Cross Correlation Matrix (DCC)

The cross-correlation is the correlation between the entries of two random vectors X and Y, while the correlations of a random vector X are the correlations between the entries of X itself, those forming the correlation matrix of X. In such a matrix, the correlations of the various temporal instances of X with itself are known as autocorrelations, and they are arranged on the matrix diagonal. Outside the diagonal, there are the cross-correlations between X and Y across the time, which assume the value between +1 and −1. We considered that the regions are correlated when such a value is greater than 0.75, and they are anti-correlated between −1 and −0.75. The cross-correlation matrix was computed by means of the Bio3d package of the R Software version 3.5.3 [43,44].

### 2.6. Hydrogen Bonds and Salt Bridges Interactions

The hydrogen bonds were computed using the gmx hbond GROMACS tool [33]. The salt bridges interactions were computed using the LigPlot Software version 2.2 [45].

### 2.7. Cluster Analysis and Binding Free Energy Estimation (MMPBSA)

The cluster analysis was performed using the gmx cluster GROMCAS tool [33], setting a cutoff of 2 Å, and the Gromos Clustering Algorithm [46] for clusters determination.

The estimation of the free energies of binding were carried out using the gmx_MMPBSA tool based on AMBER’s MMPBSA.py [47,48]. The pMHC was selected as ‘ligand’, and both the chains of the TCR were selected as ‘receptor’. The analyses were performed on 1000 frames sampled during the trajectories.

## 3. Results

### 3.1. Conformational Analysis

Due to the huge dimension of the complexes, two MD simulations of ~600 ns were performed for each system, and one MD simulation of ~700 ns for the unbound state (Tables S2 and S3).

**Figure 4:**
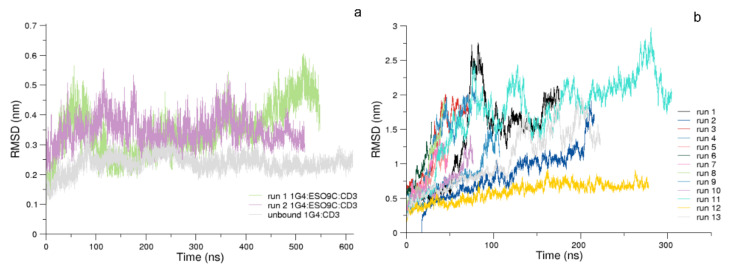
The C-*α* RMSD. In panel (**a**), the RMSD of the 1st run of 1G4:ESO9C:CD3 (lilla), the 2nd run of 1G4:ESO9C:CD3 (light green), and of the unbound 1G4:CD3 (gray). In panel (**b**), the RMSD of the 13 trajectories of the 1G4:ESO4D:CD3 complex; in all these simulations, the dissociation of the pMHC was observed, except for run 12 (gold).

Unexpectedly, in the 1G4:ESO4D:CD3 simulations, the dissociation of the HLA-A*02: peptide was observed. To assure the reproducibility of this event, 12 additional MD simulations of the system were performed (Table S3). The dissociation event was not found for the 1G4:ESO9C:CD3, where the pMHC remains bound to the TCR for the entire simulation (see Figure 4) and, thus, no further simulations were run for such a system.

The RMSDs analysis showed that the 1G4:ESO9C:CD3 and the unbound 1G4:CD3 both reach a plateau after several ns, because of the larger dimension of the complex (Figure 4). Therefore, the first 180 ns were removed from the analysis. To compare 1G4:ESO9C:CD3 with 1G4:ESO4D:CD3, the single MD run where the complex remains bound along the trajectory (Figure 4b) has been taken into account. 

**Figure 5:**
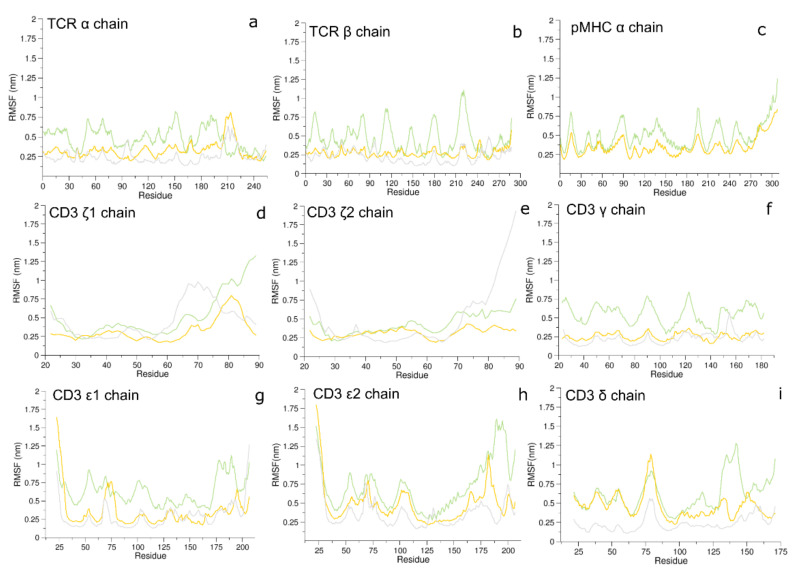
The RMSF computed on the alpha carbons. Panel (**a**,**b**): the RMSF of the TCR *α* and *β* chains, for 1G4:ESO9C:CD3 (green), 1G4:ESO4D:CD3 (yellow) and 1G4:CD3 unbound (gray). Panel (**c**), the RMSF of the *α* chain of the pMHC for 1G4:ESO9C:CD3 (green) and 1G4:ESO4D:CD3 (yellow). Panel (**d**–**i**): the RMSF of the CD3 chains for 1G4:ESO9C:CD3 (green), 1G4:ESO4D:CD3 (yellow) and 1G4:CD3 unbound (gray).

The RMSFs analysis—averaged on the trajectories where possible—of the three systems show comparable fluctuations in the CD3ζ chains only. In fact, in the other protein regions the fluctuations in the 1G4:ESO9C:CD3 system are larger than those observed in both the 1G4:ESO4D:CD3 and in the unbound systems (Figure 5).

Such differences are even larger in the TCR *α* and *β* chains and in the CD3*γ* chain.

### 3.2. Collective Motions

To double check that the systems have reached the convergence, a PCA analysis was computed on the alpha carbons, excluding the transmembrane region and the CD3 chains. The unbound trajectories as well as the trajectories of the bound states were divided into two parts and projected on the first two eigenvectors. The convergence of the system is verified if the conformations explored in the first and the second part of the trajectory are not randomly distributed. Our data show that the systems reached convergence, and in particular, a conformational change affecting the ESO9C complex was observed in the second run (Figures S1 and S2).

**Figure 6:**
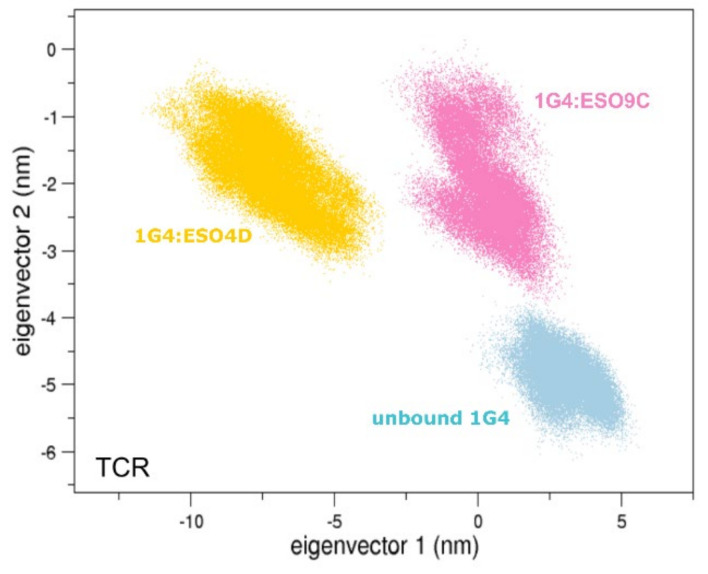
The 2D projections of TCR. The projections of the TCR alpha carbons (excluding the transmembrane regions) on the first two eigenvectors of the unbound 1G4:CD3 (lightblue), 1G4:ESO9C:CD3 (pink) and 1G4:ESO4D:CD3 (gold).

Then, to compare the motions of the simulated complexes the trajectories were concatenated to compute the common eigenvectors describing the overall motions of the unbound and bound states. Such an analysis applied to the whole TCR (Figure 6) shows that the three systems explore different regions of the essential subspace as defined by the two first eigenvectors, which describe more than 50% of the total variance of these systems (Figures S3 and S4, Table S4). 

Concerning the single TCR domain, the conformations explored by the Vαβ regions of the complexes are partially superimposed. The residues mostly affecting their motion belong to the CDR3 loops. These loops are known to be essential in the recognition of the peptide [9,10,11,12] (Figure S4).

**Figure 7:**
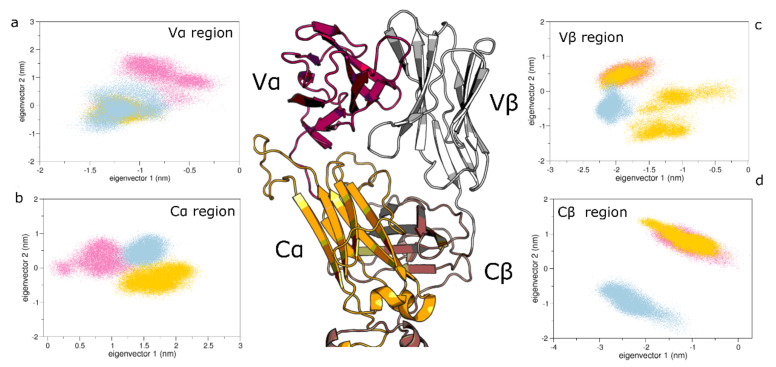
The 2D projections of the Variable (V) and Constant (C) regions of the TCR for 1G4:ESO9C (pink), 1G4:ESO4D (gold) and unbound 1G4:CD3 (light blue). Panel (**a**) the projections for the Vα region. Panel (**b**) projection for the Cα region. Panel (**c**) projection for the Vβ regions. Panel (**d**) projection for the Cβ region.

**Figure 8:**
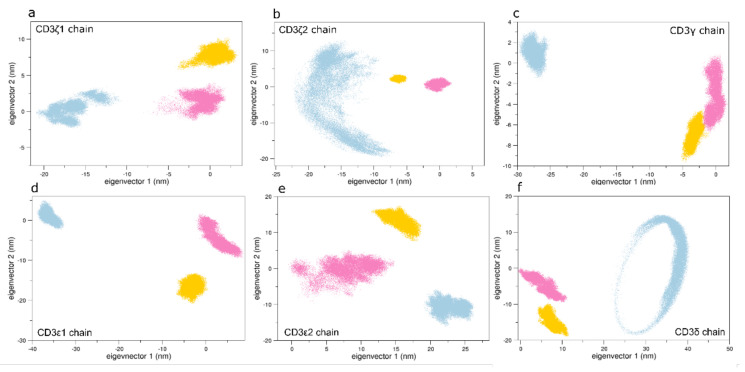
The 2D projections of the CD3 chains. The conformations of the bound states 1G4:ESO9C:CD3 (pink) and 1G4:ESO4D:CD3 (gold) discriminate from the unbound 1G4:ESO4D ones (light blue) on the first eigenvector, which describes about the 70% of the total variance of the system. Panel (**a**) projections for CD3*ζ*1 chain; panel (**b**) projections for CD3*ζ*2 chain; panel (**c**) projections for CD3*γ* chain; panel (**d**) projections for CD3*ε*1 chain; panel (**e**) projections for CD3*ε*2 chain; panel (**f**) projections for CD3*δ* chain.

On the other hand, the C*β* region of the unbound state differs from the structures sampled by both pMHC-TCR systems (Figure 7d). These data, in line with the NMR spectroscopy outcomes [13,15], was not observed in our previous study [27]. This indicates that the presence of the CD3 chains affects the conformations of the TCR*β* chain. On the other hand, the conformations of the other TCR regions are almost similar, in line with our previous simulations [34]. Projecting the CD3 conformations on the same essential subspace, it is noticed that CD3γ and CD3δ of the bound state explore similar regions with respect to the unbound one (Figure 8c,f). Here, the first eigenvector describes about 70% of the total variance of the system, thus indicating that the CD3 chains of the unbound system explore different regions of the conformational space, as described by the first eigenvector.

Finally, the binding groove alpha carbons projections (residues 1–180 of the HLA-A*0201 alpha chain, Figure 2a) reveal that similar regions are explored by the ESO9C and ESO4D systems (Figure S5).

### 3.3. Dynamic Cross-Correlation (DCC) of the Bound States

**Figure 9:**
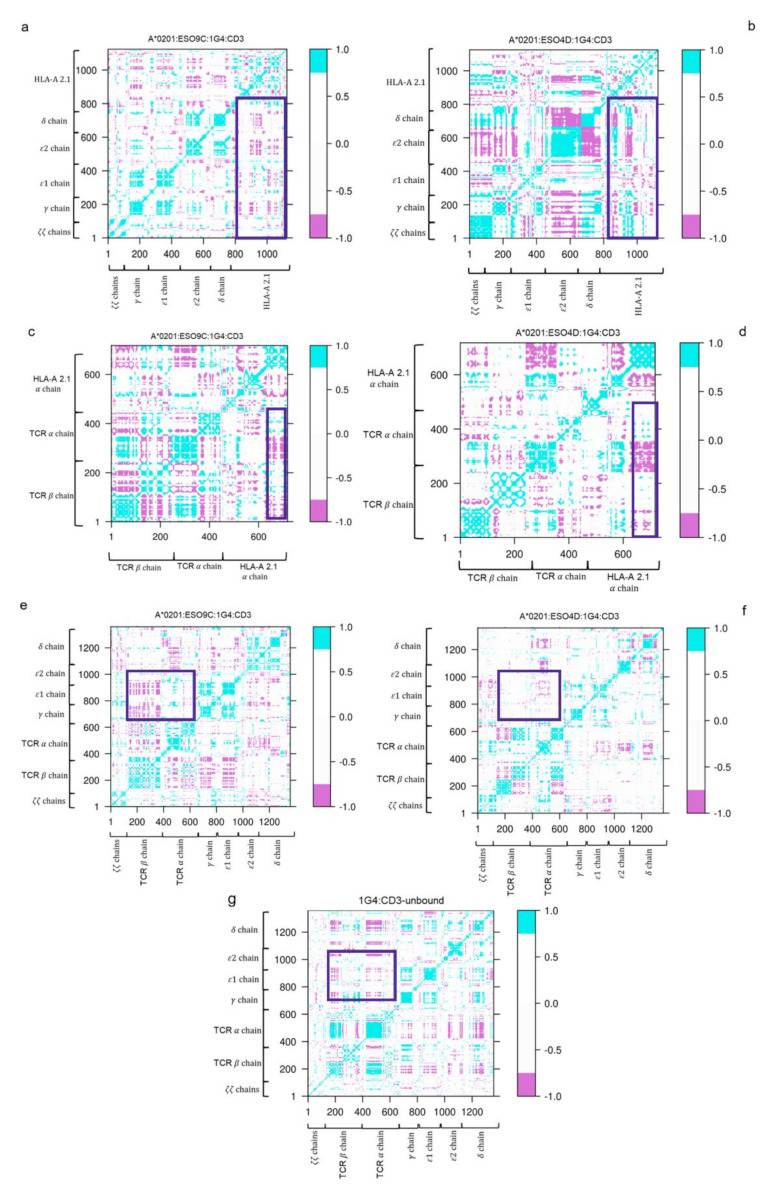
Dynamic Cross Correlation (DCC) matrices of the bound states. In panels (**a**,**b**), the DCC matrices computed for the HLA-A*02.01 and the CD3 chains in the bound states. In panels (**c**,**d**), the DCC matrices of the TCR and the HLA-A*02.01. In panels (**e**–**g**), the DCC matrices related to the TCR and the CD3 chains. The cyan regions indicate the presence of a correlation; the violet regions indicate an anti-correlation; the white regions show a low correlation.

The DCC maps of both the 1G4:ESO9C:CD3 and 1G4:ESO4D:CD3 complexes have been analyzed (Figure 9). 

Interestingly, the presence of the ESO4D peptide induces a higher correlation between the HLA-A*02.01 and the CD3 chains with respect to the ESO9C. 

However, the most remarkable differences are found in the DCC maps of the HLA-A*02.01 and *αβ* TCR regions (Figure 9c,d). In fact, the comparison of these two maps clearly shows that in the case of 1G4:ESO9C:CD3, the C*β* region motions correlate with both the *α* and *β* regions of the TCR, whereas these correlations are absent or strongly reduced in the case of 1G4:ESO4D:CD3. 

Such a behavior is also observed between the C*β* region and the CD3*ε* and CD3*γ* chains, where a striking correlation is observed in the case of 1G4:ESO9C:CD3 only.

On the contrary, in the unbound states the δ chain shows negative correlations with all the other CD3 chains. Moreover, the correlation between the C*β* region and the CD3*ε* and CD3*γ* chains—observed in 1G4:ESO9C:CD3—is less pronounced. Thus, concerning this region, such correlations are weak in the unbound state; they increase in the ESO9C model and disappear in the 1G4:ESO4D. A significative correlation between the C*β* region and CD3*ε* and CD3*γ* chains were also observed in a very recent work on the unbound TCR:CD3 complex [49]. 

### 3.4. Interface Interaction Behavior of the Bound States

**Figure 10:**
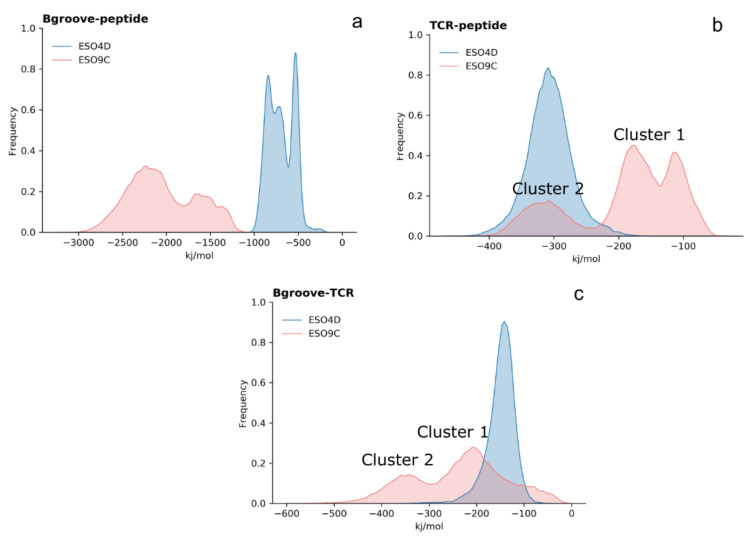
The distributions of the interaction energies of the complexes. In panel (**a**): the interaction energy between the binding groove and the peptide; in panel (**b**): the interaction energy between the TCR and the peptide; in panel (**c**): the interaction energy between the TCR and the binding groove.

To better understand the interaction behavior between the main regions at the interface between the TCR and the pMHC, their interaction energies have been analyzed (Figure 10). The corresponding distributions show more favorable interactions between the binding groove and the peptide in the ESO9C complex with respect to the ESO4D. This result, quite unexpected as the number of residues considered is almost the same, indicates that the ESO9C peptide affects the conformational behavior of the binding groove, allowing more favorable interactions between the peptide and the neighbors residues of the complex.

A similar behavior is also observed in the case of the interaction between the TCR and the binding groove (Figure 10c).

On the other hand, the same analysis on the TCR-peptide shows the opposite behavior, with the interaction energies being lower (more favorable) in the case of 1G4:ESO4D:CD3 (Figure 10b). Interestingly, the 1G4:ESO9C:CD3 interaction energy distributions between these regions, e.g., TCR, binding groove and peptide, are quite broad and show a bimodal behavior, indicating the presence of different types of interactions, in contrast to the 1G4:ESO4D:CD3 complex, where these distributions are significantly sharper. The analysis of the structures sampled along the MD trajectories shows two structural clusters (Figure S6), which are responsible for the multimodal behavior of the interaction energies of the ESO9C complex. These two clusters show an RMSD between the whole structure of 5 Å, mostly due to conformational rearrangement of the CDR3 loops.

The different interaction behavior observed between 1G4:ESO9C:CD3 and 1G4:ESO9C:CD3 explains well the unbinding events observed in the MD simulations of the latter. In fact, after a time interval between 10 and 200 ns, we observed in 12 out of 13 MD trajectories the separation of the complex (Figures S7 and S8, Table S3 and Video S1). 

To better characterize the energetic behavior responsible for the different profiles of these complexes, the analysis of the interaction at the interface between TCR and MHC I was performed (see below). To estimate the free energy of binding (ΔG_b)_ between the TCR and pMHC, the MM-PBSA approach was used, using the gmx_MMPBSA tool based on AMBER’s MMPBSA.py [47,48]. However, the difference between these ΔG_b_ is lower than the associated statistical error, thus preventing a reliable estimate of this property. This is probably due to the very large size of the molecular constructs involved in the binding process (Figure S9).

### 3.5. Hydrogen Bonds and Salt Bridges

To map the interactions between the peptide, the MHC I and the TCR, an analysis of the hydrogen bonds as well as of the electrostatic interactions was carried out (Tables S5–S7, Figures S10 and S11). Our data show that both the peptides are stabilized in the MHC I binding groove by the hydrogen bonds between the Glu63 (α1 chain) and the Tyr99 (α2 chain). As already reported in our previous work [27,50], these two residues anchor the initial part of the peptide, interacting with the a.a. residues Ser1 and Leu2 (Figure 2a and Table S5).

Moreover, the last part of the peptides (residues 8,9) is anchored to the pocket thanks to the hydrogen bonds between Asp77 (MHC I α1 chain) and Thr142 (MHC I α2 chain), in line with our previous findings (Figure 2a and Table S5) [27,50].

The main electrostatic interactions between the peptides and the binding groove are listed in Tables S6 and S7 and in Figures S10 and S11.

Finally, hydrogen bonds and salt bridge interactions were found between the peptides and the TCR CDR3 regions (Figure 2b, Figures S10 and S11 and Table S5 and Figure S6). This is in line with the fact that it is known that these loops are involved in the detection of the peptide, which then induces the triggering [9,10,11,12]. All these interactions are maintained in the two clusters of conformations found in the ESO9C system (Figure S10 and Table S6).

Interestingly, these kinds of interactions were not found between the MHC I and the TCR, suggesting a key role of the peptide in the MHC-TCR linkage. Such behavior suggests that the coupling of the motion between the MHC and TCR is mainly due to long-range interactions.

### 3.6. Solvent Exposure of the CD3 Chains

**Figure 11:**
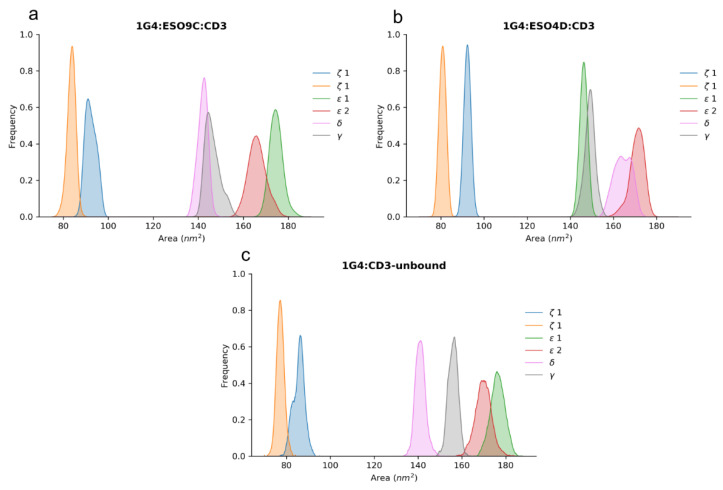
Solvent exposure of the CD3 chains. Panel (**a**) solvent exposure of the CD3 chains related to 1G4:ESO9C; panel (**b**) solvent exposure related to 1G4:ESO4D; panel (**c**) solvent exposure related to the unbound 1G4.

The solvent exposure analysis computed on the CD3 chains shows that the CD3*ζ*1 and CD3*ζ*2 chains are less exposed to the solvent in all the simulated systems (Figure 11). However, these results might be affected by the different lengths of the single chains.

Interestingly, the effect of the pMHC is to increase the solvent exposure of the CD3*ε*2 and the CD3*δ* regions with respect to the unbound state. This is in line with the NMR data, where possible allosteric effects on these chains were hypothesized [15].

## 4. Discussion

To consider the role of the CD3 complex in the conformational behavior involved in the triggering process, the first computational model of the pMHC:TCR:CD3 complex was built in a lipid environment. In this work, the MHC I (HLA-A*02.01) bound to TCR (1G4) in the presence of two different peptides, i.e., ESO9C (a.a sequence SLLMWITQC) and ESO4D (a.a. sequence SLLDWITQV), were modeled. Such systems experimentally show different values of the kinetic constants (Table 1) as well as from the ability of the peptide to induce the activation of the TCR, measured in terms of IFN*γ* release (i.e., the ESO9C induces a greater activation of the receptor compared to ESO4D [25]). 

Therefore, two MDs for the ESO9C complex and thirteen for the ESO4D complex were carried out. In addition, to compare the conformational behavior between the bound and unbound states, an MD of the unbound state of the 1G4 interacting with the CD3 complex was performed.

Contrary to our previous results [27], where the CD3 chains were not included in the simulated system, a conformational change affecting TCR C*β* region of the bound state was found. Such a change was already reported in previous experimental works [15,27], where differences in the chemical shifts have been observed upon pMHC binding, by means of NMR spectroscopy.

Similarly, we also observed that the bound and unbound conformations of the CD3 chains explored different regions of the conformational space, indicating a propagation of the signal to regions of the complex far from the pMHC:TCR interface. It is reasonable to consider such an effect as the first event able to induce TCR triggering by phosphorylation of the CD3 chains, which react to the presence of the pMHC by changing their conformational behavior.

The effects on the motion correlations observed in both the TCR C*β* region and in the CD3 chains due to the presence of the pMHC suggest that the pMHC:TCR complex is tightly regulated by allosteric effects, which propagates from the binding region—as represented by the peptide in interaction with both the TCR and the MHC I binding groove—up to the intracellular regions of the complex.

This is line with the most recent experimental works where the T-cell activation was explained by such structural-dynamic changes observed in those regions, e.g., CD3 chains and C*β*, by means of NMR spectroscopy [13]. Interestingly, a remarkable correlation was observed between the C*β* region and the CD3*ε*-CD3*γ* chains in the ESO9C system. This correlation is weak in the unbound state, and it disappears when the TCR interacts with the ESO4D peptide. Hence, the structural-dynamic coupling within C*β*, CD3*ε* and CD3*γ* chains can have a role in the triggering process, and it might contribute to conformational changes, leading to a greater exposure of the ITAMs.

In addition, the different interaction behavior in the 1G4:ESO9C with respect to the 1G4:ESO4D as well as the unbinding events observed for the latter suggests that the interactions binding groove-peptide and binding groove-TCR might play a main role in the signal propagation. That is, in the case where a minor activation of T-cells was experimentally reported, i.e., the 1G4:ESO4D complex, these interactions are less favorable with respect to the 1G4:ESO9C system, which displays a higher activation. In other words, the propagation of the signal from the TCR-pMHC interface to the C*β* region observed in the 1G4:ESO9C only and the unbinding events sampled by MD simulations of the 1G4:ESO4D might contribute to explain the role of the peptide in the different T-cells response experimentally observed.

## 5. Conclusions

In this work, we presented an extended computational study concerning the interaction of the pMHC:TCR:CD3 complex embedded in a lipid environment. Two systems, differing in the peptide only, were modeled and used to perform all-atom MD simulations. The structural and dynamical characterization of these complexes confirmed our previous results: independently from the specific peptide bound to the complex, the pMHC binding grooves explore similar conformations. Thus, this finding points out that the peptide does not directly induce conformational changes in the binding pocket, which is confined in a more rigid conformation upon the interaction with the TCR.

Comparing the bound and unbound states, conformational changes affecting the C*β* region were observed. Furthermore, coupling motions between such a region and CD3*ε*/CD3*γ* chains were found in the complex able to activate the T-cells. This might suggest a role of the C*β* region, affecting the CD3*ε*-CD3*γ* chain dynamics, in regulating the exposure of the ITAMs, the regions undergoing phosphorylation by Lck kinase.

Finally, all our results point out that an accurate modeling of the T-cell receptor in a realistic environment can shed some light on the structural-dynamic mechanism put in place by this molecular complex to trigger its response.

## Figures and Tables

**Table 1:** The simulated complexes and the corresponding experimental binding affinity data [25]. The MHC class I is the HLA-A*02:01. The mutated residues are reported in red. Activation potency of each pMHC in IFNγ release assay is presented by the EC50 value [25].

Complex with HLA-A*0201	Peptide Sequence	KD (uM)	Koff (s − 1)	t ½ (s)	Kon (M − 1 s − 1)	EC₅₀ (IFN-γ) (μg/mL pMHC)
1G4-ESO9C	SLLMWITQC	14	0.82	0.84	57 × 10 + 3	115 ± 14
1G4-ESO4D	SLLDWITQV	252	2.59	0.27	10 × 10 + 3	661 ± 85

## Data Availability

Upon request to the authors.

## Supplementary Materials

The following are available online at https://www.mdpi.com/article/10.3390/cells11040668/s1, Figure S1: The 2D projections of the unbound TCR, Figure S2: The 2D projections of the bound pMHC:TCRs, Figure S3: Cumulative variance of the first five eigenvectors of the PCA computed on the combined trajectories, Figure S4: Eigenvector components related to the PCA performed on the combined trajectories, Figure S5: Binding grooves, 2D projections, Figure S6: Cluster analysis of 1G4:ESO9C, Figure S7: Number of the ESO4D-dissociation events as a function of time, Figure S8: Distances of pMHC from the TCR for the dissociated ESO4D trajectories, Figure S9: Binding free energy profiles, Figure S10: Salt bridges between ESO9C and 1G4, Figure S11: Salt bridges between ESO4D and 1G4. Table S1: Box details of the built systems, Table S2: MD simulations performed for ESO9C:CD3 and the unbound TCR:CD3, Table S3: MD simulations performed for the ESO4D:CD3 system, Table S4: The cosine content of the first 10 eigenvectors for the PCA computed on the combined trajectories, Table S5: Hydrogen bonds between peptides, binding groove and TCRs, Table S6: List of the salt bridge interactions between ESO9C peptide, the binding groove and the TCR, Table S7: List of the salt bridge interactions between ESO4D peptide, the binding groove and the TCR, Video S1: Dissociation event of the HLA-A*02:01:ESO4D from the TCR:CD3 complex.
