# Supplementary material for: The Full Model of the pMHC-TCR-CD3 Complex: A Structural and Dynamical Characterization of Bound and Unbound States
Source: Cells. 2022 Feb 14;11(4):668. doi: 10.3390/cells11040668 (PMC8869815; doi:10.3390/cells11040668)
Supplement: Supplementary file 1 [file cells-11-00668-s001.zip › SI/SI_paper.pdf]

Supplementary Information

1. Systems details

Table S1. Boxes details of the built systems. The number of water molecules and ions added to each complex are listed. Na+ and Cl- ions were added at physiological concentrations to neutralize the system. The average periodic boundary condition (PBC) cells dimensions are reported on the right.

System	# Water molecules	# Na+ ions	# Cl- ions	# Tot atoms	PBC cell (x y z) nm
1G4:ESO9C:CD3	271231	1449	984	1049641	15 x 15 x 44
1G4:ESO4D:CD3	265616	1451	984	1014647	15 x 15 x 44
unbound 1G4:CD3	142639	739	504	543861	15 x 15 x 21

Table S2. MD simulations of the ESO9C:CD3 and the unbound TCR:CD3.

Number of runs	Unbound 1G4:CD3	ESO9C:CD3
1	~700ns	~600ns
2	//	~600ns

Table S3. MD simulations of the ESO4D:CD3 system. The time of the dissociation events are reported in the right column.

Number of runs	Total ns performed	Detachment time
1	71 ns	10ns
2	28 ns	18ns
3	47 ns	23ns
4	216 ns	24ns
5	49 ns	28ns
6	80 ns	30ns
7	42 ns	32ns
8	175 ns	65ns

9	77 ns	70ns
10	107 ns	83ns
11	304 ns	115ns
12	222 ns	190ns
13	501 ns	420ns

2. Convergence analysis

Figure S1. **The 2D projections of the unbound TCR**. The projections of the TCR alpha carbons (excluding the transmembrane regions) on the first 2 eigenvectors of the unbound 1G4:CD3 are shown. The black (orange) dots represent the trajectory projections of the 1st (2nd) half part of the equilibrated trajectory.

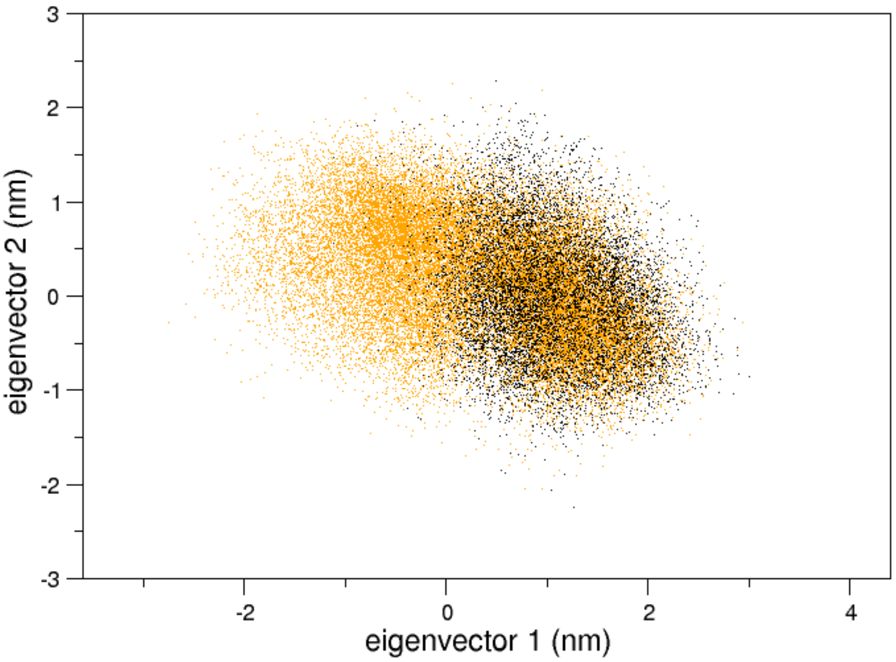

Figure S2. **The 2D projections of the bound pMHC:TCRs**. The projections of the all-alpha carbons (excluding the transmembrane regions and the CD3 chains) on the first 2 eigenvectors of 1G4:ESO9C (panel a-b) and 1G4:ESO4D (panel c) are shown. For 1G4:ESO4D the analysis was carried out on the undissociated

system. The black dots represent the projections related to the 1st half part of the equilibrated trajectory; the orange ones represent the projections related to the 2nd part of the trajectory.

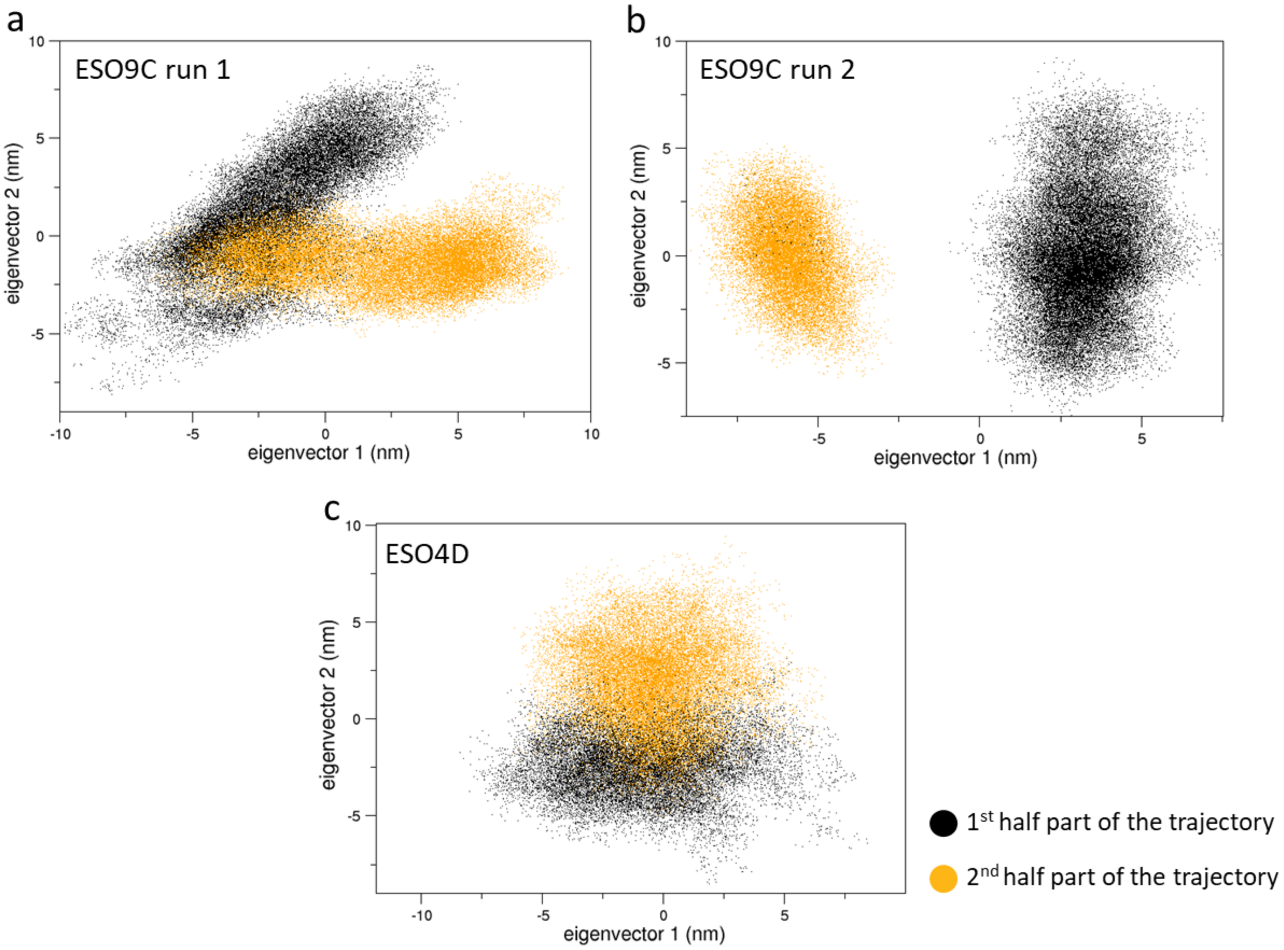

Figure S3. **Cumulative variance of the first 5 eigenvectors of the PCA computed on the combined trajectories.** The first two principal components explain more than 50% of the variance.

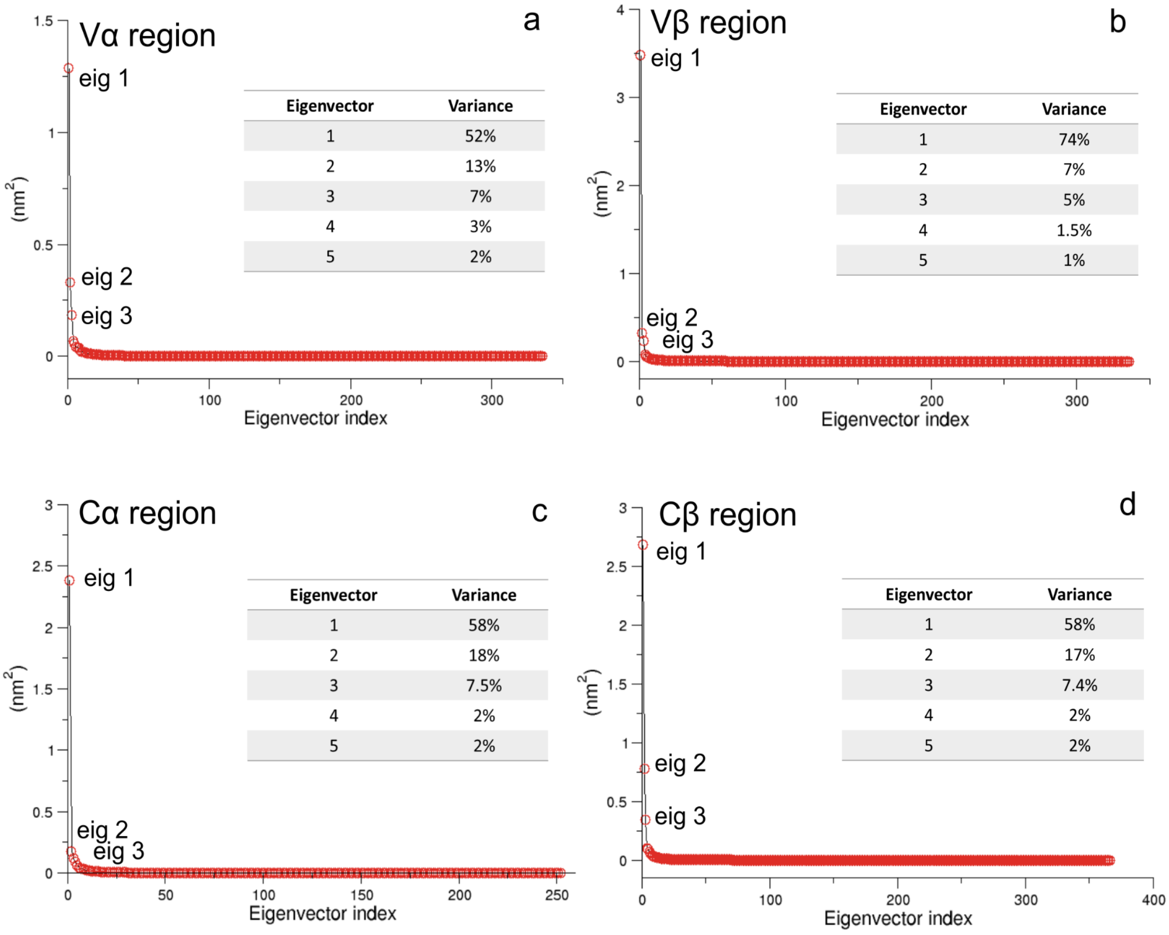

Table S4. The cosine content of the first 10 eigenvectors for the PCA computed on the combined trajectories. When the cosine content is closed to 1, the largest fluctuations are not connected with the potential, but with random diffusion.

Bgroove	TCR V*α*	TCR V	TCR C*α*	TCR C	Bgroove
**Cosine content**	0.33	0.31	0.24	0.23	0.32

Figure S4. Eigenvector components as obtained by the PCA on the combined trajectories. The residues which contribute most to defining the direction of the eigenvector are labeled (panel a) and visualized (panel b). The residues most affecting the motion of the variable regions belong to the CDR3 loops.

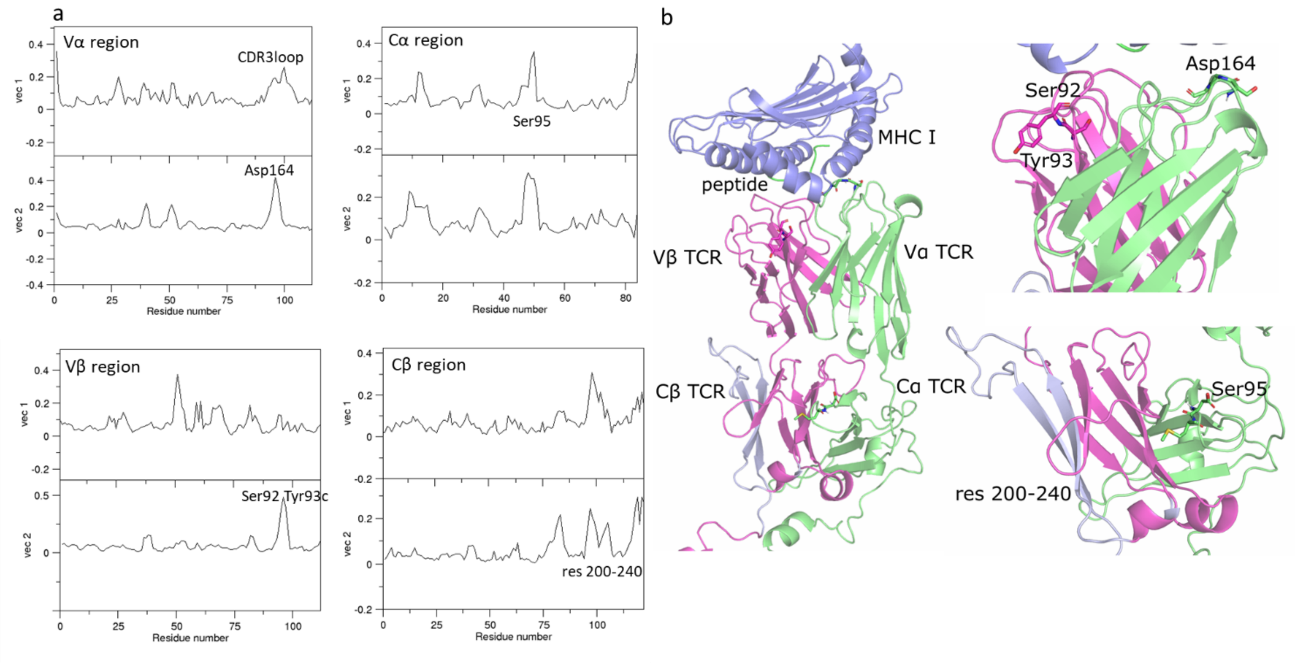

3. Projections of the binding groove

Figure S5. **Binding grooves 2d projections**. The projections of the HLA-A*02.01 binding groove alpha carbons of the bound states (ESO9C:1G4:CD3 in blue, ESO4D:1G4:CD3 in red) on their essential subspace as defined by the first two eigenvectors.

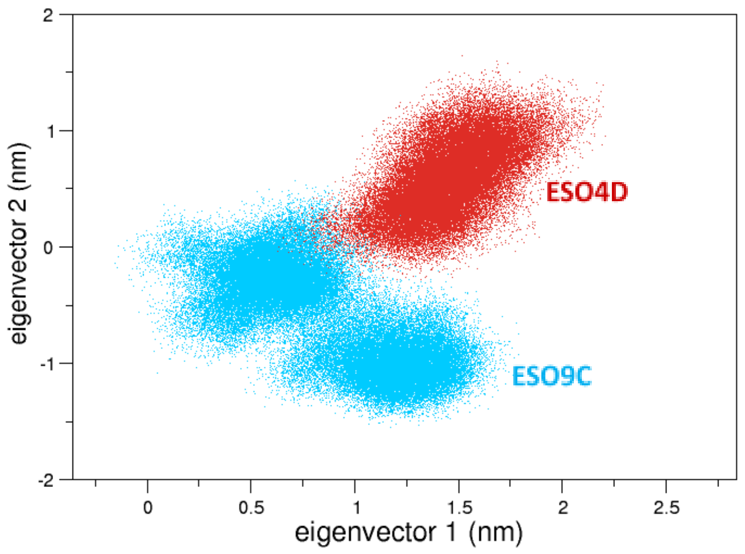

4. Cluster analysis for ESO9C systems

Figure S6. **Cluster analysis of 1G4:ESO9C**. The analysis was performed using the gmx cluster Gromacs tool, setting a cutoff of 2 Å, and the Gromos Clustering Algorithm for clusters determination. Two clusters with an RMSD between the whole structure of 5 Å were identified. The CDR3 regions (brown and violet) which mostly interact with the peptide (see below 'Hydrogen bond and salt bridges') show the typical conformational variability related to these loops.

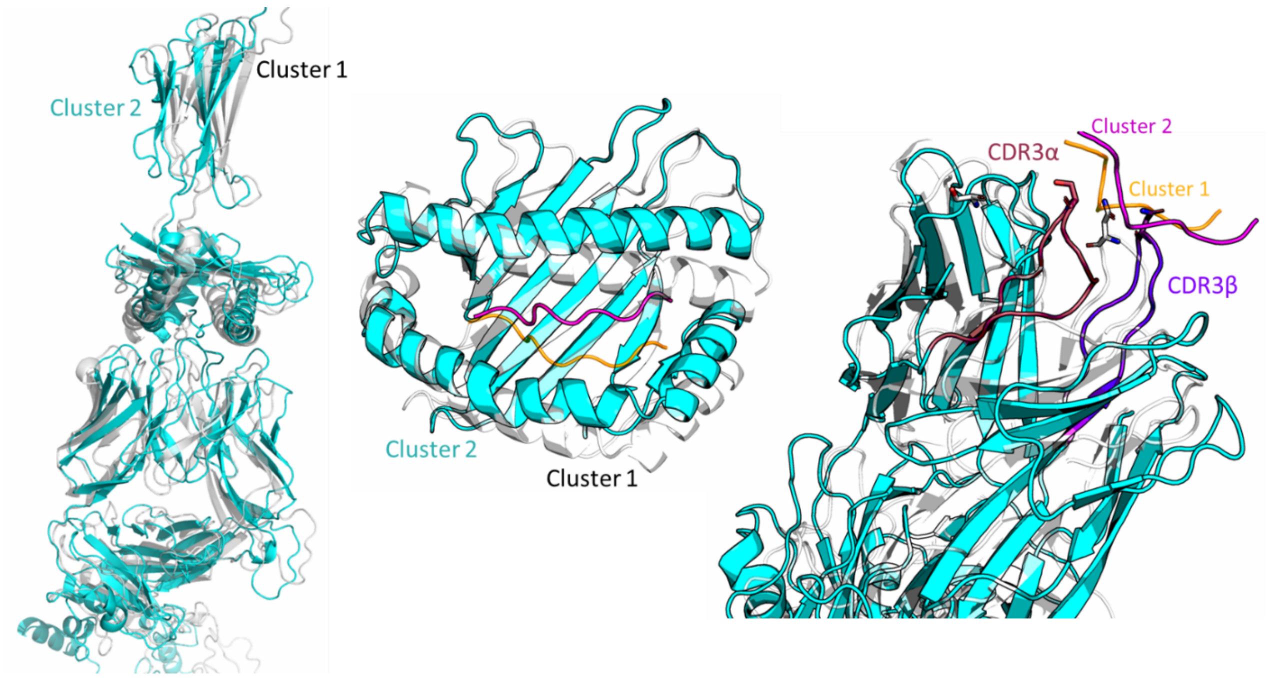

5. ESO4D dissociation events

Figure S7. **Number of the ESO4D dissociation events as a function of time**. Black points represent the time of the dissociation events of pMHC/TCR:CD3-complex. The points were fitted imposing a first-order kinetic behavior (red line). The correlation coefficient is 0.96.

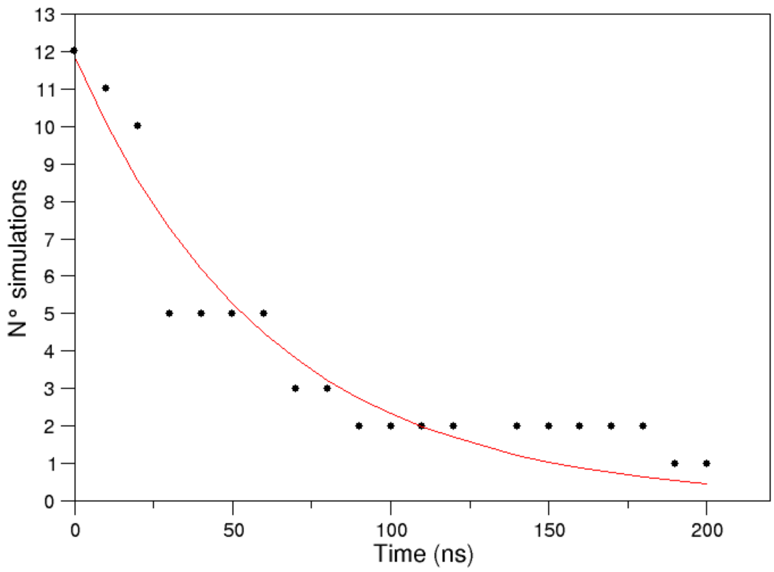

Figure S8. **Distances of the pMHC from the TCR for the dissociated ESO4D trajectories**. The distances were computed between the center of mass (COM) of the pMHC and the COM of the variable regions of the TCR. Then, a running average was performed every 500 points to smooth the curves. The last ns, where the pMHC is completely dissociated from the TCR, were removed from the analysis.

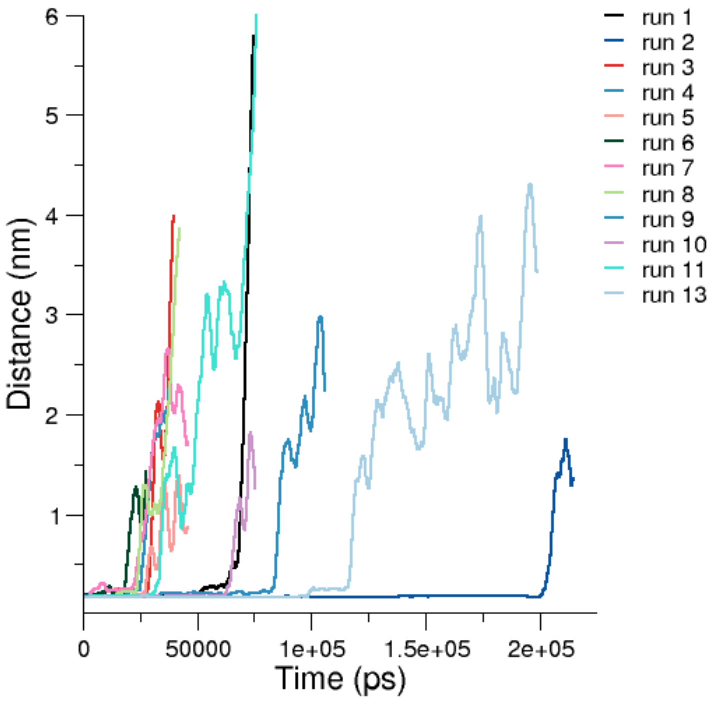

6. Binding free energy estimation - MMPBSA

Figure S9. Binding free energy profiles. The binding free energies (with error bars) were estimated using the gmx_MMPBSA tool based on AMBER's MMPBSA.py [47-48]. The pMHC was selected as 'ligand', and both the chains of the TCR were selected as 'receptor'. The analysis was performed on 1000 frames sampled during the trajectories.

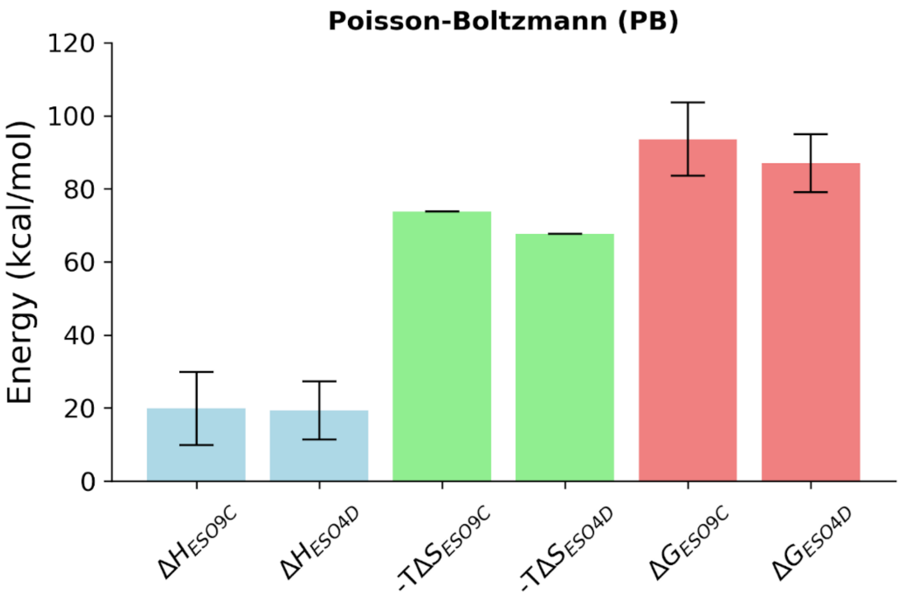

7. Hydrogen bonds and salt bridges

Table S5. Hydrogen bonds between peptides, binding groove and TCRs. Hydrogen bonds between the MHC I binding groove (Bgroove) and the peptide were found only for the ESO9C system. Hydrogen

bonds are established between the CDR3 loops of the TCR and the central residues of both peptides. No hydrogen bonds were found in the other regions, neither between the TCR and the binding groove.

1G4:ESO9C	Bgroove	TCR CDR3α	TCR CDR3β	1G4:ESO4D	Bgroove	TCR CDR3α	TCR CDR3β
Ser1	Glu63/Tyr159			Ser1	Glu63/Tyr159		
Leu2	Tyr99			Leu2	Tyr99		
Leu3				Leu3			
Met4		Tyr120		Asp4		Tyr120	
Trp5		Pro113	Asn118	Trp5		Pro113	Val116
Ile6				Ile6			Gly117/Asn118
Thr7			Gly117	Thr7			Asn118
Gln8	Asp77/Trp147			Gln8	Asp77		
Cys9	Thr143/Lys146			Val9	Thr143		

Figure S10. **Salt bridges between ESO9C and 1G4**. The salt bridge interactions between the peptide and the TCR were performed using the LigPlot Software [45]. The analysis was carried out on the average structure obtained from the cluster analysis. The interactions are summarized in Table S6.

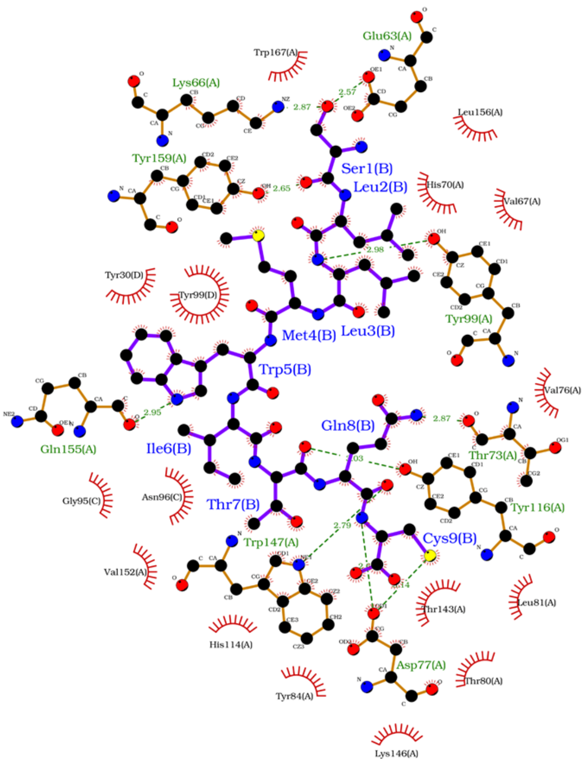

Cluster 1

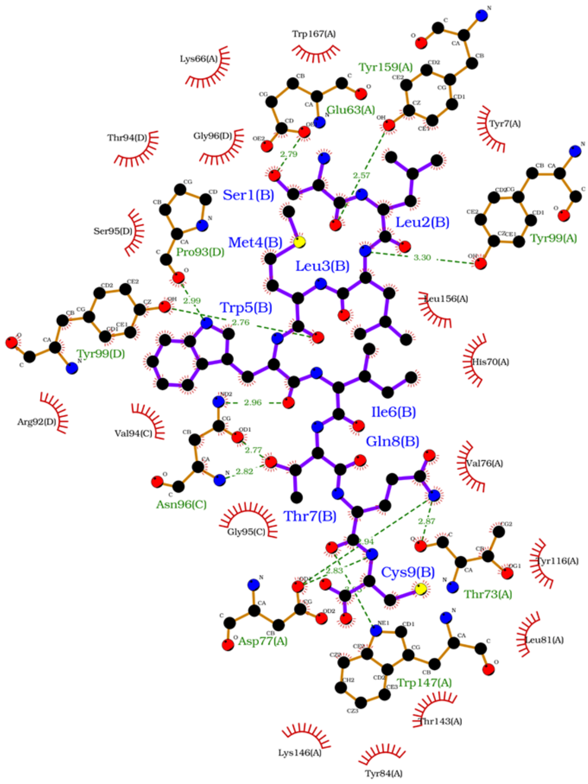

Cluster 2

Table S6. List of the salt bridge interactions between ESO9C peptide, the binding groove and the TCR. The table summarized the data obtained by the LigPlot analysis.

1G4:ESO9C Cluster 1	Bgroove	TCR CDR3α	TCR CDR3β	1G4:ESO9C Cluster 2	Bgroove	TCR CDR3 α	TCR CDR3β
Ser 1	Lys66			Ser 1			
Leu 2	Tyr159			Leu 2	Tyr159		
Leu 3	Tyr99			Leu 3	Tyr99		
Met 4				Met 4		Tyr119	
Trp 5	Gln155			Trp 5		Pro113	
Ile 6				Ile 6			Asn118
Thr 7	Tyr166			Thr 7			Asn118
Gln 8	Thr73/Trp14 7			Gln 8	Thr73/Trp14 7		
Cys 9	Asp77			Cys 9	Asp77		

Figure S11. **Salt bridges between ESO4D and 1G4**. The salt bridge interactions between the peptide and the TCR were performed using the LigPlot Software [45]. The analysis was carried out on a representative structure extracted from the undissociated trajectory. The interactions are summarized in Table S7.

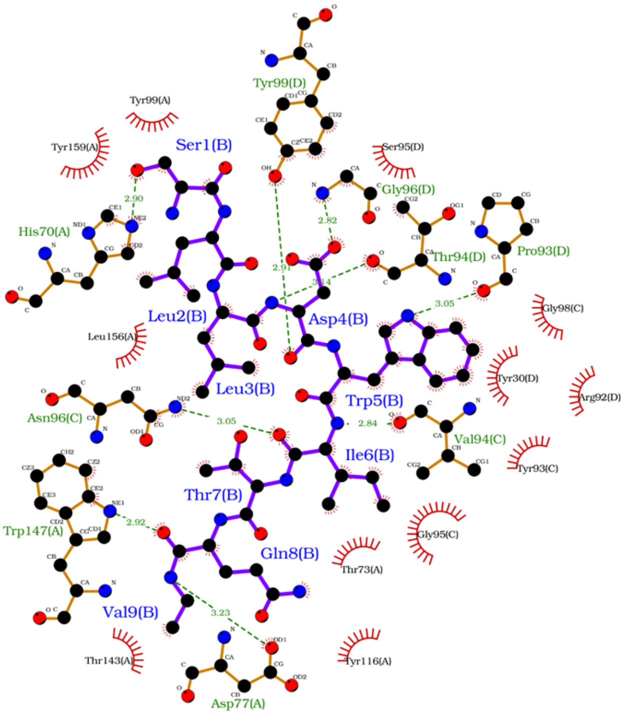

Table S7. List of the salt bridge interactions between ESO4D peptide, the binding groove and the TCR. The table summarized the data obtained by the LigPlot analysis.

1G4:ESO4D	Bgroove	TCR CDR3α	TCR CDR3β
Ser1	His70		
Leu2			
Leu3			
Asp4		Gly96/Thr94/Pro93	
Trp5			
Ile6			Asn96
Thr7			

Gln8	Trp147		
Val9	Asp77		
